# Combination of classical and statistical approaches to enhance the fermentation conditions and increase the yield of Lipopeptide(s) by Pseudomonas sp. OXDC12: its partial purification and determining antifungal property

**DOI:** 10.3906/biy-2106-59

**Published:** 2021-12-14

**Authors:** Vivek CHAUHAN, Vivek DHIMAN, Shamsher Singh KANWAR

**Affiliations:** 1 Department of Biotechnology, Himachal Pradesh University, Summer Hill India

**Keywords:** Fermentation, optimization, purification, TLC, antifungal activity, statistical evaluation

## Abstract

Around 200 different lipopeptides (LPs) have been identified to date, most of which are produced via *Bacillus* and *Pseudomonas* species. The clinical nature of the lipopeptide (LP) has led to a big surge in its research. They show antimicrobial and antitumor activities due to which mass-scale production and purification of LPs are beneficial. Response surface methodology (RSM) approach has emerged as an alternative in the field of computational biology for optimizing the reaction parameters using statistical models. In the present study, *Pseudomonas* sp. strain OXDC12 was used for production and partial purification of LPs using Thin Layer Chromatography (TLC). The main goal of the study was to increase the overall yield of LPs by optimizing the different variables in the fermentation broth. This was achieved using a combination of one factor at a time (OFAT) and response surface methodology (RSM) approaches. OFAT technique was used to optimize the necessary parameters and was followed by the creation of statistical models (RSM) to optimize the remaining variables. Maximum mycelial growth inhibition (%) against the fungus Mucor sp. was 61.3% for LP. Overall, the combination of both OFAT and RSM helped in increasing the LPs yield by 3 folds from 367mg/L to 1169mg/L.

## 1. Introduction

In recent times, Lipopeptides (LPs) have gained a lot of attention from the science community respective of their vast applications. Lipopeptides have turned out to be one of the most important secondary metabolites produced by microorganisms leading to growing research interest in them. With more than 200 different LPs identified to date, they are structurally diverse compounds (Kumar et al., 2021). The high structural variability is the resultant of frequently occurring amino acid substitutions. This characteristic feature of LPs in turn gives them the ability to decrease interfacial and surface tension. Structurally, they are low molecular weight compounds that consist of a fatty acid acyl chain (hydrophobic) attached to a peptide head (hydrophilic) (Mukherjee et al., 2021). The fatty acid chain does not exceed more than 17 carbons in length, whereas the number of amino acids ranges anywhere between 7 and 35. Most documented LPs are produced from *Pseudomonas*- (Proteobacteria) and *Bacillus*- (firmicutes) strains. Other strains reported to produce LPs are *Streptomyces* (Nielsen et al., 2000) and some fungal strains (Verma et al., 2019). 

Vastly studied LPs obtained from *Bacillus* strains are characterized as iturin, surfactin, lichenysin, and fengycin, and those produced by *Pseudomonas* strains are tensin, surfactin, viscosin, massetolid, arthrofactin, pseudodesmin, syringomicin, xantholysin, and pseudofactin. In most cases, the difference among the structures of different lipopeptide (LP) is due to the rearrangement of amino acids or the addition or removal of carbon atoms in the fatty acid chains (Koumoutsi et al., 2004). These LPs find applications in different sectors, including pharmaceuticals, agriculture, textile, and petroleum. Studies show that many LPs can act as excellent antimicrobial and antifungal agents against different pathogenic micro-organisms (Chauhan et al., 2021). Thus, LPs can help in the production of biomedicines against ever-evolving pathogenic strains which are antibiotic-resistant (Matsui et al., 2020). LPs have also proven their worth as a xenobiotic compound that can be used to degrade petroleum products and help in bioremediation (Zhu et al., 2020).

Different LPs can be produced upon alteration of nutrient conditions in the growth environment (Morikawa et al., 2000). Many nitrogenous and carbon sources have been reported to affect the production of different LPs mainly iturins, surfactins, and fengycins (Vigneshwaran et al., 2021). Nowadays, a variety of cheap counterparts such as rice bran, soybean, potato-peels, molasses, etc. are used for LPs production to tackle the production cost. In addition, various metal ions as Mn^2+^ and Fe^2+^ are known to enhance LP production (Rangarajan et al., 2012). In a study, addition of manganese to the growth medium increased LPs yield from 0.33 to 2.6 g/L (Matsui et al., 2020). Further, the presence of MnSO_4_, FeCl_3_, and ZnSO_4_ in the growth medium for *Bacillus* subtilis increased surfactin production (Zhu et al., 2020).

The major limitation in LPs production is high production cost and low yield. Diverse applications of LPs have bided scientists to harness ways to enhance its production. This is done by optimizing different growth parameters to achieve enhanced LP production. Conventionally one factor at a time (OFAT) method is used in which a single parameter or factor is examined at a time while keeping other parameters to constant. OFAT has certain shortcomings as it is a time-consuming process, requires more data for analysis, and studying the interaction between different factors or variables is quite cumbersome. Due to these limitations of OFAT many different approaches are looked upon to provide the desired results. Response surface methodology (RSM) is one such approach that is explored by scientists to reach an optimal value obtained by interaction among different variables. RSM is a statistical tool that comprises statistical and mathematical techniques for model fitting, preparing an experimental design, optimization, and validation of a few selected physicochemical factors. By using different statistical tools available in RSM, an experiment can be designed using the desired variable or factors. In RSM, different variables act as an input, and their interaction will result in an optimized output (Nair, 2013). Central composite design (CCD) and Plackett–Burman design (PBD) are the two most noteworthy experimental designs used for optimization in a microbial fermentation process (Khusro et al., 2016). PBD acts as the (first) screening step of RSM process. Here, all the variables are screened and selected on the basis of their ability to positively affect the optimization process. Selected factors later serve as an input to create CCD where interaction among them is studied to get optimal results. As only minimum process knowledge is required for RSM, it is both cost- and time-effective (Palvannan et al., 2010).

A noteworthy limitation to models developed through RSM is that it is accurate only for a narrow range of inputs process parameters and the development of higher-order RSM models requires a larger time, numerous experiments to be performed, and they are costly. Keeping this limitation in view a combination of OFAT and RSM techniques was used to determine LP production for the present study. The present study is aimed at enhancing the LP production in fermentation broth from *Pseudomonas* sp. OXDC12 is a strain isolated from the soil sample in HPU, Shimla. Interaction of different independent variables were analysed using both OFAT and RSM approach to maximize LP production by the *Pseudomonas* strain.

## 2. Materials and methods

### 2.1. Chemicals, microorganisms, and culture media:

The bacterial strain OXDC12 used in the study was isolated from the field soil of spinach cultivation and was identified as a *Pseudomonas* sp. by 16s rDNA gene sequencing (MN336228) (Shruti et al., 2021), and the identifier was a mucor sp. isolated from capsicum annum plant and identified using 18s-RNA (Meena et al., 2018). All chemicals used for the study were of analytical grade Sigma-Aldrich (Steinheim, Germany). The solid media used for antifungal experiments contained Luria-Bertani agar for bacteria (LBA: yeast extract, 5g; peptone, 10g; agar, 18g; NaCl, 10g; and distilled water, 1L), and potato dextrose agar (PDA: agar, 18g; glucose, 20g; potato, 200g; and distilled water, 1L) was used for fungi. The liquid medium used for fermentation tests was LB medium (prepared with the same components as present in LBA but without agar). To activate the strain OXDC12 single colonies of the strain were transferred from plates to 30mL liquid LB activation medium in 100mL flasks as the seed culture. The flasks were incubated with shaking at 160 rpm for 14h at 37°C.

### 2.2. Time profile of the growth of Pseudomonas sp. OXDC12 and antifungal activity:

*Pseudomonas* sp. strain OXDC12 was initially inoculated on LB agar slant and then transferred to 500mL (2L flask) of LB medium by shaking at 130 rpm at 37°C for 24h. A 5mL equivalent fraction of the culture was collected every two hours from 0 to 78h. Optical density (OD) was read at each time point. Thereafter, to access the antimicrobial activity, 5mL of the culture was centrifuged at 13,500g for 1 min to obtain the cell-free supernatant. The antifungal test was conducted over mucor sp. using 100μL of this cell-free supernatant by the well-diffusion method (Tagg, 1971). For this, 24h-old spore of test pathogens cultures in potato dextrose broth (PDB) at 30°C was spread over Potato dextrose agar (PDA) plate. Test culture was then poured in the wells created using agar hole puncture 8mm diameter and checked for % inhibition after 3 days (Meena et al., 2018).

### 2.3. Extraction and mass concentration calculation of LPs:

*Pseudomonas* sp. strain OXDC12 from a seed culture (6h) was incubated in a 250mL Erlenmeyer flask containing 100mL of LB medium with shaking at 180 rpm for 24h at 30°C. After cultivation, the culture was centrifuged at 10000 rpm for 15 min. and the cells pellet was discarded. The pH value of the cell-free supernatant was adjusted to 2.0 using 6M HCl and stored overnight at 4°C for acid precipitation (Yao et al., 2012). Further, the precipitate was collected by centrifugation at 9500g for 15min at 4°C. The supernatant was discarded, and the pallet/residue was extracted using the minimal amount of methanol under shaking conditions. The crude product was tested for LP presence and antimicrobial activity. Methanol was evaporated from the crude LP in an oven at 60°C (Cao et al., 2012). The residue was weighed and used to calculate the mass concentration.

### 2.4. Assays for lipopeptide(s):

#### 2.4.1. Quantification of peptide and lipid contents:

The different assays were performed to check the peptide and the lipid moiety of the extracted LPs. Peptide quantification was done using the Bradford test (Bradford, 1976), while the presence of lipid moiety in the extract was checked using the Sudan IV test (Patel et al., 2015). Sudan IV (Red) was added in methanol to make a 1mg/mL stock solution. A total of 1mL of the sample was taken and five drops of Sudan IV stock solution were added to it. In the presence of lipid moiety, color of Sudan IV changes from red to orange. 

#### 2.4.2. Thin-layer chromatography (TLC) analysis of LP:

A 5μL of sample (LP) sterilized with 0.22-micron membrane was applied onto a TLC plate (Silica gel 60/UV254, SDFCL, thickness: 0.2 mm and 1 cm × 25 cm). TLC plate was then transferred into the solvent/mobile phase. The mobile phase consisting of chloroform: methanol: water (65:25:4) was used in the analysis. The TLC plate was developed by uniformly spraying the TLC plate with ninhydrin solution (0.25% in ethanol) and was placed in an oven at 110ºC for 20 min. This was then used to detect the peptide moiety of LP. Similarly, the lipid moiety of the LP was detected by uniformly spraying the TLC plate with water and analyzing it under UV light (Razafindralambo et al., 1993). *R* *_f_* value of the extracted LP was calculated by the following formula); 

*R* *_f_* = Distance traveled by solute from the origin (cm) / Distance traveled by solvent from the origin (cm)

#### 2.4.3. Analysis of the antifungal activity of LPs:

Antifungal activity assay was performed for isolated lipopeptide(s). The assay was performed using agar well diffusion method (Tagg, 1971) on freshly prepared potato dextrose agar (PDA) Petri-plates. The test fungus culture (Mucor sp.) was inoculated in the middle of the PDA plates and to the peripheral wells (diameter 6 mm), methanol (30µL) was loaded in the control petri-plate whereas lipopeptide preparation extracted using methanol (30µL) was loaded aseptically in the test petri-plate. These petri-plates were then incubated at 30ºC and growth inhibition (%) was recorded against the fungal pathogen after 1 and 3 days, respectively. The following equation was used for the calculation of the zone of inhibition:

% = Dc – Dt / Dc  100

Where Dt: Average diameter of mycelial colony treated with LPs. Dc: Average diameter of control mycelial colony.

### 2.5. Optimization of fermentation conditions to enhance LPs production:

#### 2.5.1. Conventional one factor at a time (OFAT) approach:

Initial tests were performed using LB medium containing no extra carbon or nitrogen sources at pH = 6, 37°C agitated at 130rpm. One factor at a time (OFAT) approach was employed to optimize various physio-chemical parameters like culture medium, inoculum size, inoculum age, initial pH value, nitrogen source, carbon source, and effect of different metal ions for enhancing the LP production. Different nitrogenous sources (peptone, ammonium sulphate, urea, sodium nitrate, yeast extract, ammonium nitrate, beef extract, and ammonium chloride) at a concentration of 1% (w/v) were added to the production media separately to study their effect on lipopeptide(s) production. Similarly, different carbon sources (glucose, sorbitol, lactose, galactose, maltose, sucrose, mannitol, fructose, and starch) were also studied for optimal LP production. The effect of pH (4, 5, 6, 7, 8, and 9) and agitation rate (50, 80, 100, 130, 160, 190, and 210rpm) was tested separately for the production of LP in the fermentation broth. The effect of different metal ions (Fe^3+^, Zn^2+^, Mg^2+^, Na^+^, Mn^2+,^ and K^+^) was checked. The yield of the LP obtained in each case was determined and recorded which further helped in assessing different parameters for designing the RSM models. The best response/factor providing optimal LP yield served as the center point around which the RSM model was designed. For each setup, three parallel tests were conducted.

### 2.6. Response surface methodology (RSM) analysis for the statistical optimization of LP production by Pseudomonas sp. strain OXDC12:

OFAT optimization method was followed by the RSM approach to enhance LP production in the fermentation broth. RSM analysis was done by combining two different model designs i.e., the Plackett–Burman Design (PBD) and Composite Center Design (CCD).

#### 2.6.1. Plackett–Burman 

The experimental design for PDB is based upon the 1st order model which assumes that there is no interaction amongst fermentation medium constituents and the parameters under study (xi).

Y=*β*0+∑*β* *_ixi_*, (1)

where, Y = estimated target function

*βi*= was the regression coefficient

For the construction of the PBD model, eight production variables were used which had an independent effect on the fermentation broth. The screening of these variables was based upon responses at two levels, i.e. minimum and maximum. In general, PBD is a fractional factorial design, which is used to measure the difference between the averages of observations at the maximum (+1) and the minimum level (–1) of the factors (Diwaniyan et al., 2011; Nair, 2013). For this study, PBD was prepared using eight selected parameters (beef extract, glucose, production time, pH value, centrifugation rate, centrifugation time, temperature, and MnSO_4_). Software Design expert 12.0 was used to prepare the experimental designs which suggested 12 different experimental runs with contrasting values for the selected parameters. The study was carried in 12 runs, and the observations were fed into the same software (Design expert) for statistical analysis.

As PDB is only used as a screening tool, it cannot be used as the only design tool to efficiently carry out the RSM optimization process. Hence, the screened variables were further selected for the CCD study. 

#### 2.6.2. Central composite design:

Central composite design (CCD) was then employed to measure the relation between selected variables to further assist in the optimization of LP production. CCD measures the interdependence of variables where the experiment is designed on the basis of 2n factorial and 2n axial runs. Centre runs are used to calculate experimental error, which helps in proofreading the created design. Here, 2n factorial was coded by +1 and –1 level and each independent variable/factor was investigated for these two levels. Test runs are proportional to the number of variables (n) and increase rapidly when the number of variables increases. Thus, the experiment was designed using the CCD model for optimizing the LP growth from *Pseudomonas* sp. OXDC12. Four variables (beef extract, production time, glucose concentration, and production temperature) were screened out as beneficial for LP production and were used for experimental design. The effect of 30 runs was generated and recorded for further analysis. Both designing of the experiment and data analysis was done using Design Expert, Version 12.0.0 (Stat-Ease Inc., Minneapolis, MN). The three-dimensional surface (3D)-plots were also obtained for the CCD which gives the information about the main effect and interactive effects of the independent variables used in the experiment (Meena et al., 2018).

#### 2.6.3. Proofreading of PBD and CCD:

Proofreading is necessary to check the authenticity of experimental runs obtained from PBD and CCD designing techniques for LP optimization in the fermentation broth. ANOVA and the lack of fit test are used to check the authenticity of the experimental design. The desired model is one that has a significant value for the ANOVA test and a non-significant value for the Lack of fit test. Also, the perturbation plot created in the case of CCD helped in the validation of the test.

### 2.7. Statistical analysis:

All experiments were done in triplicate, and the average concentration of LP was considered as a response. The statistical analysis of OFAT data was done using Microsoft Excel (MS Office 2019), whereas ANOVA and lack of fit test to prove the credibility of PDB and CCD were done (Gangadharan et al., 2008) using Design-Expert software package (version 12.0.0, State-Ease Inc., USA).

## 3. Results

### 3.1. Generation of growth curve vs. antifungal activity curve

**Figure 1:**
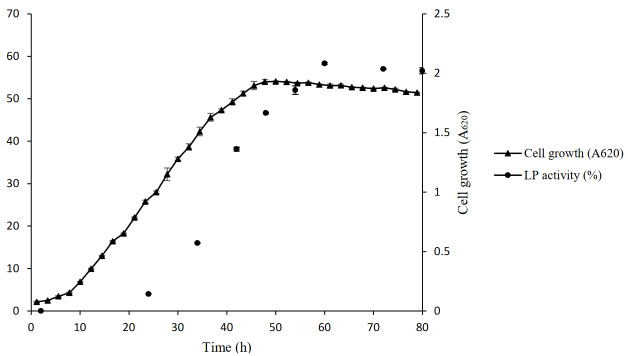


The growth curve of *Pseudomonas* sp. OXDC12 is shown in Figure. 1. The bacteria grew well in LB medium, with the logarithmic phase appearing at 14h to 22h. Using the same LB medium, the antifungal activity at different time points in the culture was measured, and the relevant curve was generated (Figure 1). The antifungal activity peaked at 60h (58.31 ± 0.24) and was in the stationary phase of the culture. Based on the generated curve, 60h old cultures were considered as optimum for detecting the antifungal activity for the crude LPs. 

### 3.2. Analytical tests for LPs confirmation and Partial purification

**Figure 2:**
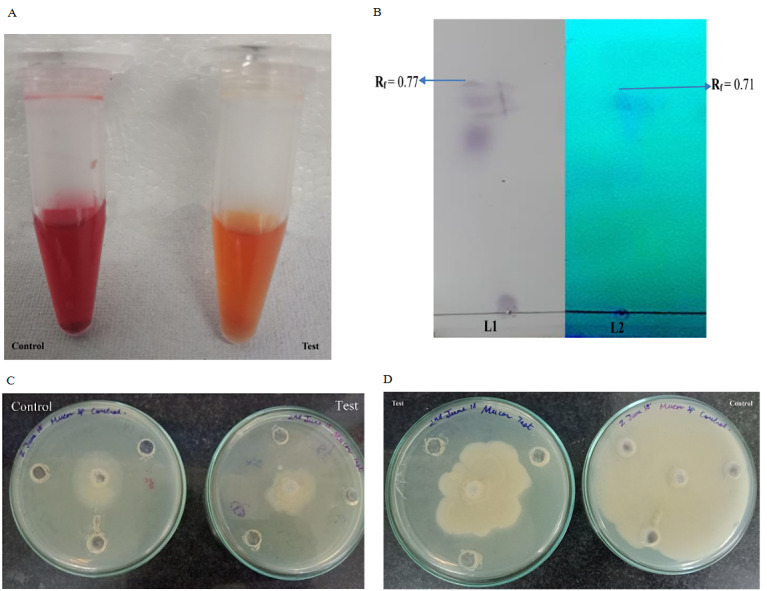


Crude LPs sample was subjected to Bradford analysis to detect protein content and presence of protein moiety. 1.21mg/mL of protein content was found in the crude sample. Sudan IV test confirmed the lipid moiety in the crude sample. Initial screening was followed by TLC analysis. A large spot was visible on the TLC plate when sprayed with water and examined under UV having *R* *_f_* values of 0.77 and 0.71 (Figure 2a, 2b). When seen under normal light it appeared to be white indicating the lyophilic nature of the compound. Further, LPs presence was confirmed when the other half of the plate was tested with ninhydrin for the presence of amino acids. A brown spot emerged with the same *R* *_f_* value when the plate was uniformly sprayed with ninhydrin solution (0.25% in ethanol) and placed in an oven at 110°C for 20 min.

### 3.3. Antifungal test

Antifungal activity was tested for LPs against mucor strain. No distinctive difference was found in the maximum activity achieved in both cases. Maximum mycelial growth inhibition (%) against the fungus *Mucor* sp. (Figure 2c, 2d) was 61.3% for LPs. 

### 3.4. OFAT optimization

**Figure 3:**
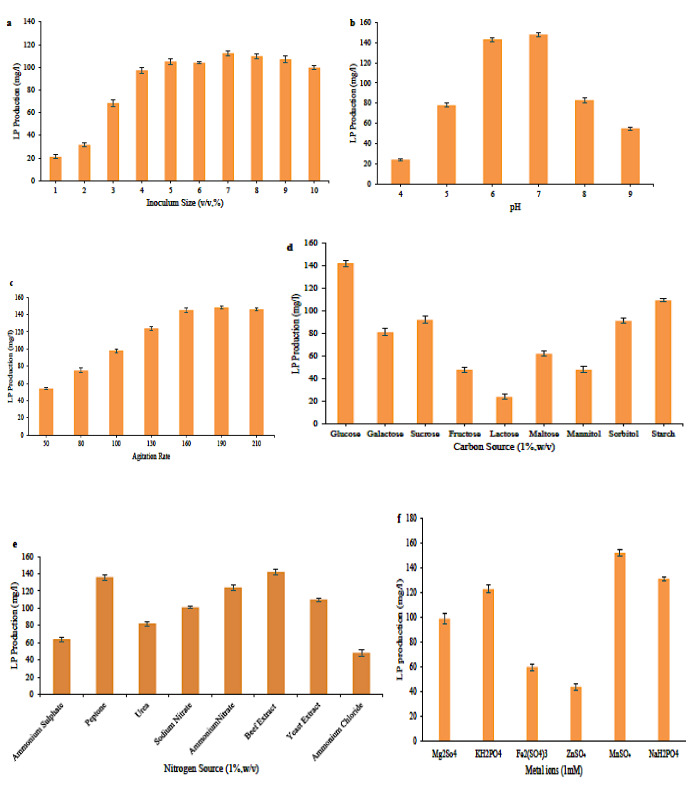


Prior to the RSM approach, the OFAT technique was used to optimizing essentials parameters affecting fermentation conditions. Effects of culture conditions, including inoculum size, initial Ph value, agitation rate, carbon source, nitrogen source, and metal ions were investigated (Figure 3). Glucose (142 ± 2.68 mg/mL) was considered as the best carbon source (Figure 3d), whereas beef extract (143 ± 3.22 mg/mL) emerged as the best nitrogen source (Figure 3e). Mn^2+^ (98.66 ± 4.04 mg/mL) showed an enhancement in LPs production in the fermentation broth (Figure 3f). It was worth noting that LPs production changes drastically when moving away from neutral pH. Initial pH value of 6 to 7 (148.66 ± 1.86 mg/mL) showed maximum production (Figure 3b). Eight-hour old inoculum at 6% v/v showed the best production (112.33 ± 2.23 mg/ml, Figure 3a). LPs productions increased with an increase in agitation rate to a point (160rpm, 145.33 ± 3.14 mg/mL) after which it attained constant and did not show any further increase (Figure 3c).

### 3.5. RSM approach

3.5.1. Plakett-Burman experimental design

**Figure 4:**
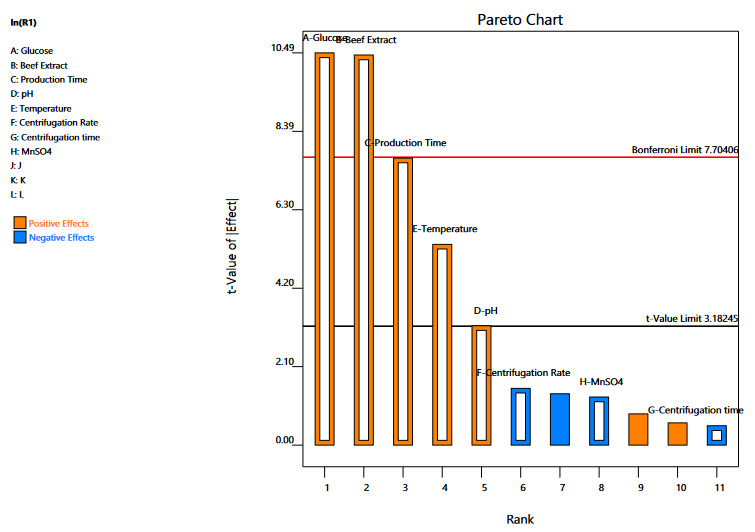


Based upon the OFAT approach effect of eight independent variables (beef extract, glucose, production time, pH value, centrifugation rate, centrifugation time, temperature, and MnSO4) was observed on the production of extracellular LPs from *Pseudomonas* sp. OXDC12. A 12 run model was created using the Plackett-Burman design, which showed the yield in a range of 146-623mg/L for different runs (Table 1). Upon analysis of Plackett-Burman design using Pareto chart (Figure 4), it was observed that five factors (beef extract, glucose, production time, production temperature, and pH value) showed a positive effect in enhancing LPs production in the fermentation broth (Table 1). Usually, a model with a p-value of <0.05 is significant. To measure the authenticity of the model ANOVA was performed and the P-value of positive variables (beef extract, glucose, production time, production temperature, and pH value) was 0.0001, whereas for the whole model (8 variables) was 0.0037 (Table 2). The difference between Predicted R² and the Adjusted R² was less than 0.2.

**Table 1:** Plakett–Burman experimental design for evaluating the influence of various independent variables on LPs production via Pseudomonas sp. OXDC12.

Run	Glucose extract (g/100mL)	Beef extract (g/100mL)	Production time (h)	pH (mM)	Temperature °C	Centrifugation rate (g)	Centrifugation time (min)	MnSO4	Response(mg/L)
1	5	5	24	7	40	20000	10	0.5	526
2	5	0.5	90	7	25	20000	20	5	324
3	5	5	90	6	25	9500	20	0.5	498
4	0.5	0.5	24	7	25	20000	20	0.5	146
5	5	5	90	6	40	20000	20	0.5	625
6	5	5	24	6	25	20000	10	5	314
7	5	0.5	24	6	40	9500	20	5	289
8	0.5	0.5	24	6	25	9500	10	0.5	138
9	0.5	5	90	7	25	9500	10	5	361
10	5	0.5	90	7	40	9500	10	0.5	462
11	0.5	0.5	90	6	40	20000	10	5	214
12	0.5	5	24	7	40	9500	20	5	281

**Table 2:** Statistical analysis of RSM moldels.

Sr. No	Test name	F- value	p-value	Predicted R²	Adjusted R²
	ANOVA-PBD (for positive {5} variables)	70.22	<0.0001**	0.9215*	0.9692*
	ANOVA-PDB (for all {8}variables)	54.40	0.0037**	0.8838*	0.9749*
	ANOVA-CCD	15.48	<0.0001**	0.7064*	0.8749*

#### 3.5.2. Central composite design (CCD)

Based upon the results of Plakett–Burman analysis four variables (beef extract, glucose, production time, and temperature) were considered for central composite design (CCD). These variables were tested for optimum level and their combined effect in enhancing the LPs production. CCD model with 30 different runs was prepared for analysis of the variables (Table 3). The responses obtained from the experimental runs served as an input for the design matrix, and the predicted response by the design matrix was presented (Table 3). From the responses, it was concluded that four variables working together positively affect the LPs production and at the concentration of beef extract 2.75% (w/v), glucose 2.75% (w/v), temperature 32.5°C, and the production time of 57h showed the highest yield (1168mg/L) of LPs. 

**Table 3:** Central composite design (CCD) response for selected variables.

Run	Beef extract (g/100mL)	Glucose extract (g/100mL)	Temperature (°C)	Production time (h)	Response (mg/L)
1	0.1	2.75	32.5	57	923
2	5	0.5	40	90	689
3	7.25	2.75	32.5	57	756
4	2.75	2.75	32.5	57	1168
5	5	5	25	24	412
6	2.75	2.75	32.5	8	256
7	5	0.5	40	24	522
8	5	5	40	90	766
9	0.5	5	40	24	389
10	0.5	0.5	25	24	345
11	2.75	7.25	32.5	57	689
12	0.5	5	40	90	672
13	0.5	5	25	90	482
14	2.75	2.75	17.5	57	98
15	0.5	0.5	40	24	355
16	2.75	2.75	32.5	57	980
17	0.5	5	25	24	367
18	2.75	2.75	32.5	57	1102
19	2.75	2.75	32.5	123	886
20	5	5	25	90	554
21	2.75	2.75	47.5	57	178
22	2.75	2.75	32.5	57	1145
23	5	5	40	24	456
24	2.75	0.1	32.5	57	926
25	5	0.5	25	90	498
26	2.75	2.75	32.5	57	1124
27	0.5	0.5	25	90	459
28	0.5	0.5	40	90	733
29	5	0.5	25	24	482
30	2.75	2.75	32.5	57	1167

ANOVA of CCD results was performed, and four process orders were observed by the design expert model. Quadratic process order proved to be the best and the same was processed for further analysis. The ANOVA of quadratic regression model demonstrated that the model was highly significant, which was evident from the Fisher’s F test (F model, mean square regression/mean square residual = 16.45) with a very low probability value [(P model >F) = 0.0001]. The model fit was expressed using a coefficient of determination, R2, which was 0.8742 for the model indicating 87% of the variability in the responses can be explained by this model. Adjusted R2 and Predicted R2 values had a difference of less than 0.2.

**Figure 5:**
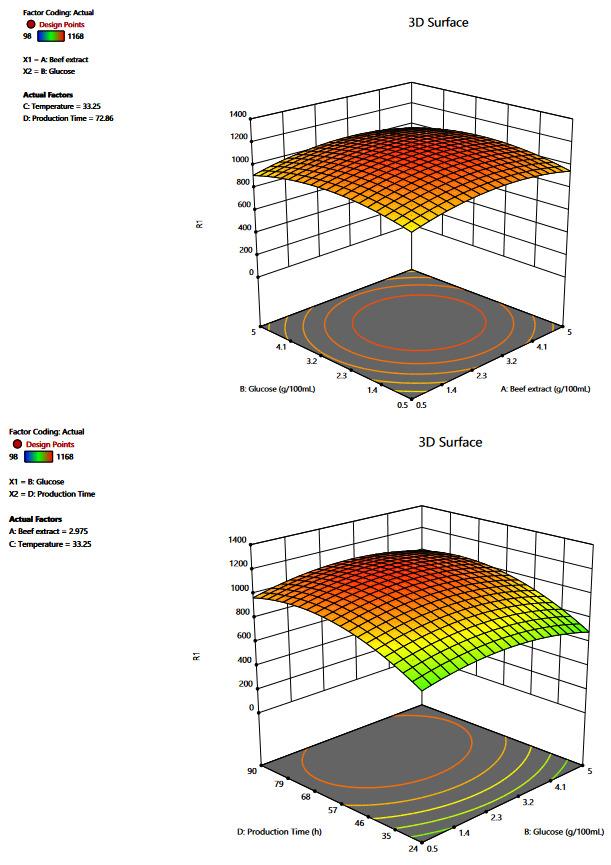

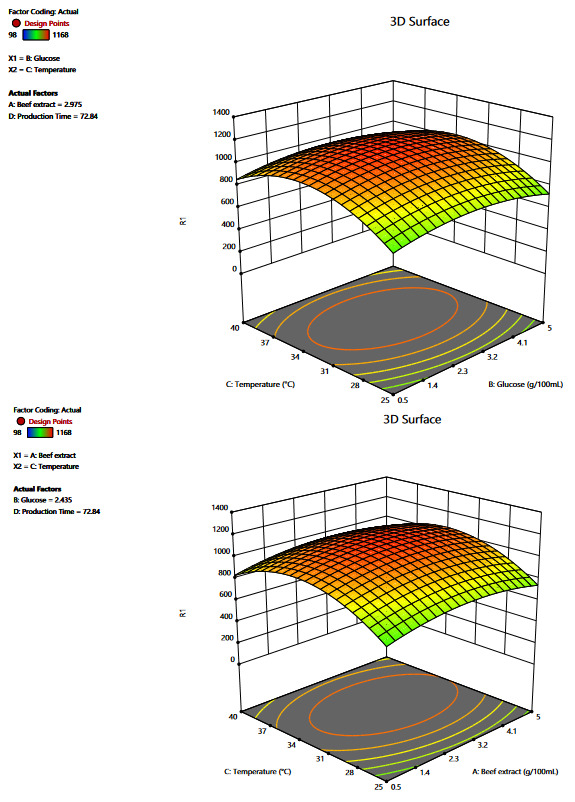


RSM approach of media optimizations is based upon the fact that different variables interact with each other to produce the best possible outcome. The interaction between the variables was studied using a 3D response surface plot (Figure. 5). 3D response model is generated from the regression analysis keeping two factors at constant and changing the other two factors with different concentrations. Using this plot, optimum levels and the interaction between variables could be understood. Four variables interacted with each other to give the best result, i.e. LPs production when the concentration of beef extract, glucose, production time, and production temperature were 2.97mg/100mL, 2.43mg/100mL 70h and 33°C, respectively (Table 4, Figure. 5)

**Table 4:** Summary of different models used in the LPs production.

Model Used	Factors optimized	LPs Production (mg/mL)
OFAT	Inoculation size	112.33 ± 2.23
	Agitation rate	145.33 ± 3.14
	Carbon source (Glucose)	142 ± 2.68
	Nitrogen source (Beef extract)	143 ± 3.22
	Metal ion (Mn2+)	98.66 ± 4.04
	pH value (6 to 7)	148.66 ± 1.86
Placket–Burman	beef extract, glucose, production time, pH value, centrifugation rate, centrifugation time, temperature, and MnSO4	625
CCD	beef extract, glucose, production timetemperature	1168

## 4. Discussion

Time and again it has been concluded that LPs have crucial applications in the environmental, agricultural, food, and pharmaceutical fields, efficient production of LPs is critical (Maksimov et al., 2020). Hence, increased heed is being paid to the quantitative and qualitative analysis of lipopeptides. Though, numerous literature is present for *bacillus* LPs a few known studies have shown effective LPs obtained from *Pseudomonas* sp. In the present study, an attempt has been made to extract LPs from *Pseudomonas* sp. OXDC12 and enhance its production using a combination of OFAT and RSM techniques. The strain was isolated from the soil, and it was identified as *Pseudomonas* sp. OXDC12 using 16S rDNA sequencing using nucleotide sequence homology and phylogenetic analysis.

The Bradford analysis and the Sudan IV test confirmed the presence of protein and lipid moiety respectively, in the crude sample (Smyth et al., 2010; Fang et al., 2014). However, TLC analysis is used in many past studies as an efficient method for the purification of the LPs (Das et al., 2008; Alajlani et al., 2016). In this study, TLC was done which confirmed the presence of LPs. *R* *_f_* value of 0.77 and 0.84 was observed. *R* *_f_* values ranging from 0.68 to 0.88 have been obtained in many past studies for LPs (Sivapathasekaran et al., 2010; Geissler et al., 2017). LPs are known to possess antimicrobial activity against various pathogenic fungi and bacteria. In the present study antimicrobial activity was tested for LPs. LPs in many different studies have been found to have good antibiotic activity against pathogenic microorganisms like *Sclerotinia sclerotiorum, Staphylococcus aureus*, and *Pseudomonas aeruginosa* (Fang et al., 2014). Purified LPs obtained from *Pseudomonas* sp. DSS73 showed antifungal activity against *Serratia marcescens *(Anderson et al., 2003). LPs obtained from *Pseudomonas* sp. CMR12a was successful in inhibiting the growth of *R. solani*. A study concluded that different types of LPs obtained from Pseudomonas sp. are capable of providing antimicrobial activity against many common pathogens (Raaijmakers et al., 2010; Geudens et al., 2018). A novel LPs isolated from *Pseudomonas* sp. UCMA 17988 showed antimicrobial resistance against *Listeria monocytogenes*, *Staphylococcus aureus*, and *Salmonella enterica *(Schlusselhuber et al., 2020).

OFAT approach was pursued for optimization with a view to develop a suitable and economical system for LPs production. Optimization of components of fermentation broth by OFAT approach involves changing one independent variable while keeping other factors constant. By using this process, several important factors were optimized. The pH value of 6–7 showed maximum production of LPs, which was in accordance with the previous studies where *pseudomonas *showed maximum production of LPs in the pH value ranging from 6 to 8 (Zhao et al., 2014; Biniarz et al., 2018).

Glucose enhanced the LPs production when added to fermentation broth as a carbon source (Hmidet et al., 2017). Many previous studies have shown that LPs production is vastly affected by the addition of a carbon source. A medium enriched with glucose (10.0g/L) showed maximum LPs produce (439.0mg/L) by *B. subtilis* ATCC 21332 (Fonseca et al., 2007). A glucose concentration of 2.5g/L was found optimum for production of lipopeptide biosurfactant from *B. subtilis* MTCC 2423 (Eswari et al., 2016). Pseudofactin (PF) lipopeptide from* Pseudomonas fluorescens* BD5 showed that the addition of nitrogen sources [Leu, Glu, amino acid mixture and (NH4)_2_SO_4_], as well as citrate and succinate as the sole carbon sources, resulted in increased production up to 120-fold, i.e. 1187 ± 13.0 mg/L (Biniarz et al., 2018).

Amongst nitrogen sources, beef extract elevated the produced amount of LPs in the fermentation broth. Nitrogenous sources like yeast extract and beef extract have enhanced LPs production when present in high concentrations in the broth (Biniarz et al., 2018). Different studies have highlighted the importance of metal ions in enhancing the LPs yield when added to the fermentation broth. For this study, the effect of different metal ions was tested and Mn^2+^ (1mM) showed positive results. The study was in accordance with many previous studies where Mn^2+^ have effectively increased LPs yield when added to the fermentation broth (Janek et al., 2016). In a study, maximum production of LPs was obtained from *Pseudomonas aeruginosa* S2 in presence of MgSO_4_ and FeSO_4_ (Chen et al., 2007). For the production of an extracellular secondary metabolite, it is important for the fermentation broth to be in motion. Hence, the rate of change in agitation rate was measured. LPs productions increased with an increase in agitation rate to a point (160rpm) after which it attained constant and did not show any further increase. This observation was quite similar to a study where agitation rate after reaching a thrashed point did not affect LPs production (Zhao et al., 2014). 

Even though the OFAT system is economical, it often fails to locate a region of optimum response model. This is due to the fact that in the OFAT system combined effects of factors on the response are not measured (Zhao et al., 2014). The RSM approach was, thus, applied as an alternate statistical tool, which helped in the evaluation of combined effects of all independent variables in a fermentation process, which may have resulted due to their interaction with each other. In the present study, eight variables (beef extract, glucose, production time, Ph value, centrifugation rate, centrifugation time, temperature, and MnSO_4_) were considered for the RSM approach based upon results obtained in OFAT optimization. The RSM approach is a two-step process; firstly Plakett-Burman experimental design is used to screen out the most important variables. This model is used as a screening tool where LPs yield-enhancing variables were screened out based upon their interaction with each other in the medium. Based upon the 12-run model showing the yield ranging from 138–625mg/L five variables (beef extract, glucose, production time, production temperature, and pH value) showed a positive effect in LPs production. The remaining three variables (Mn^2+^, centrifugation rate, and centrifugation time) showed a neutral to slightly negative effect when in LPs production. Zheng et al. (2013) used RSM to improve the lipopeptide production from *Bacillus* *subtilis* NEL-01 strain. A five-level three-factor CCD was employed to determine the effects of temperature, pH value, and culture cycle, determining that the maximum lipopeptide yield (1079.56mg/L) can be achieved at 34.81°C and for a 49.26h culture cycle. Pareto-Chart analysis helped in concluding the result of Plakett-Burman experiment. The second phase of RSM is to thoroughly examine the role of screened-out variable by use of the CCD model. Here, four variables (beef extract, glucose, production time, and production temperature) that were highly effective in enhancing LPs yield were optimized using a 24 factorial CCD design. Deepika et al. (2016) observed the following significant values of optimized conditions: Karanja oil (23.85g/L), sodium nitrate (9.17g/L), pH value (7.8), which yielded an average LPs production of 5.9072g/L at 48h, and 37°C temperature on *Pseudomonas aeruginosa *strain KVD-HR42. The maximum yield achieved using RSM was obtained in the fermentation broth containing yeast extract (2.75 mg/L) and glucose (2.75mg/L) when subjected to fermentation for 60 h at 32.5°C. In the present study, a maximum yield of 1168 mg/L was achieved. Rocha et al. (2007) were able to optimize lipopeptide yield in *Pseudomonas aeruginosa* (ATCC 10145) of 3860mg/L using cashew apple juices (1g/L) and peptone (5g/L) as carbon and nitrogen sources respectively. Wu et al. (2008) achieved Rhamnolipid yield of 8.63g/L with effective use of NaNo_3_ in *P. aeruginosa* EM1. Paul M (2020) enhanced the production of xylanase from *Pseudomonas mohnii* using RSM.

No such system can be used in the biological process if not backed by statistical studies (Myers et al., 2016; Lee et al., 2019). In order to check the authenticity of the optimization process, both stages of the RSM process were statistically tested using ANOVA. An ANOVA test is used to find out if the results of an experimental or a survey are significant or not (Kim et al., 2017). Both models were significant for ANOVA and nonsignificant for lack of fit test, which is desired for assuring the authenticity of an experimental model. Overall, using the combination of both OFAT and RSM helped in increasing the LPs yield by 3 folds from 367mg/L to 1169mg/L.

## 5. Conclusion

The main aim of the study was to find an alternative time and cost-effective approach to increase the yield of important biomolecules. RSM has emerged as an important tool in scientific-analytical studies. In combination, OFAT and RSM served as an important duo to bring down the optimization cost and give reliable results. In the present study, OFAT was used as an initial model for optimization. For RSM two models (Plakett–Burman and CCD) were used. The maximum produce using Plakett–Burman design was 526mg/L, and four factors were selected for further optimization. The maximum produce of biomolecule (LP) obtained in CCD design was 1168mg/L, which was nearly 3-folds in amount to the initially obtained value. The highly effective nature of the process encourages researchers to use it for the production and activity optimization of different biological molecules. In the future, a standardized approach can be made where a combination of classical approaches (OFAT) and statistical approach (RSM) can be used as an efficient method for the optimization process (production and activity) of biological molecules.

## Funding

This work has been funded by Council for Scientific and Industrial Research, New Delhi, under a CSIR-NET Senior Research Fellowship [File No. 09/237(0161)/2017-EMR-1] awarded to one of the authors (VC).
